# Homology modeling and global computational mutagenesis of human myosin VIIa

**DOI:** 10.15406/japlr.2021.10.00364

**Published:** 2021-03-04

**Authors:** Annapurna Kuppa, Yuri V Sergeev

**Affiliations:** Ophthalmic Genetics and Visual Function Branch, National Eye Institute, National Institutes of Health, Bethesda, United States

**Keywords:** Usher syndrome type 1B, Myosin VIIa, molecular modeling, atomic structure, genetic mutations, computational mutagenesis, inherited eye disease, genotype-to-phenotype

## Abstract

Usher syndrome type 1B (USH1B) is a genetic disorder caused by mutations in the unconventional Myosin VIIa (MYO7A) protein. USH1B is characterized by hearing loss due to abnormalities in the inner ear and vision loss due to retinitis pigmentosa. Here, we present the model of human MYO7A homodimer, built using homology modeling, and refined using 5 ns molecular dynamics in water. Global computational mutagenesis was applied to evaluate the effect of missense mutations that are critical for maintaining protein structure and stability of MYO7A in inherited eye disease. We found that 43.26% (77 out of 178 in HGMD) and 41.9% (221 out of 528 in ClinVar) of the disease-related missense mutations were associated with higher protein structure destabilizing effects. Overall, most mutations destabilizing the MYO7A protein were found to associate with USH1 and USH1B. Particularly, motor domain and MyTH4 domains were found to be most susceptible to mutations causing the USH1B phenotype. Our work contributes to the understanding of inherited disease from the atomic level of protein structure and analysis of the impact of genetic mutations on protein stability and genotype-to-phenotype relationships in human disease.

## Introduction

Usher syndrome (US) is a recessively inherited genetic disorder and the name given to a group of autosomal disorders that can result in damage to the visual and audio-vestibular systems.^1^ In the United States, the prevalence was found to be as high as 1 in 6000. US is clinically divided into three categories based on the severity of hearing problems and whether vestibular dysfunction is present or absent: Type 1, Type 2, and Type 3,^2^ with Type 1 being the most severe with loss of hearing and the onset of Retinitis pigmentosa (RP). RP leads to vision loss and further degeneration of the retina can lead to blindness.^3^ The hearing loss is usually congenital, and RP is progressive and first noticed in early childhood to the middle teenage years. A total of 13 genes have been previously associated with US, of which *CDH23, PCDH15, USH1G, MYO7A, USH1C*, and *CIB2* affect Usher syndrome type 1B (USH1B)4\.^4^ The cell is supported by a framework of cytoskeleton that is made up of microtubules (tubulin), intermediate filaments, and microfilaments (actin). Myosins are the most common group of proteins found in eukaryotes, which are motor proteins that are actin-based and are responsible for various functions including cytokinesis, production of force, and motility within the cells.^5^ Unconventional myosins, including MYO7A, are expressed in the eye and the inner ear, where they play a role in nourishing the retina and in the development and maintenance of stereocilia, respectively. Retinal pigment epithelium is maintained through melanosomes that are carried by MYO7A molecules.^6^

**Figure 1:**
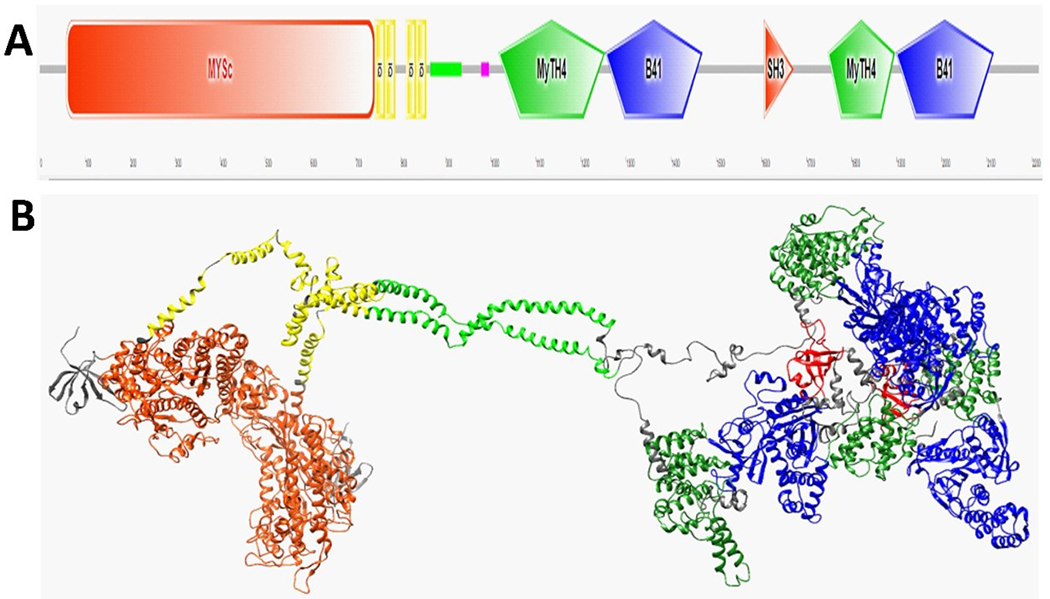
A schematic view of human MYO7A and the homology model of the human MYO7A homodimer are shown. (A) The domain structure of MYO7A predicted using SMART (http://smart.embl-heidelberg.de/). Motifs from left to right: motor domain (MySc, red); IQ motifs (yellow); coiled-coil region (green); low complexity area (magenta); MyTH4 domain (green); FERM1 domain (B41, blue); SH3 (orange), MyTH4 (green); and FERM2 domain (B41, blue). (B) A ribbon homology model of MYO7A homodimer is shown. Domains colored similarly to that of Panel A. Domain sizes are shown in Table 2.Also, the residues 632–639 are in the actin-binding region, residues 158–165 are involved in binding to ATP, and the residues 858–935 are in the single alpha-helix region formed by charged residues.

Human MYO7A is associated with the *MYO7A* gene, located on chromosome 11q13.5.^3^ A domain organization of the MYO7A showed in Figure 1A. The protein consists of a motor domain, followed by the neck region consisting of five calmodulin-binding IQ repeat motifs (CamIQ), coiled-coil domain, and the tail that is composed of Myosin tail homology 4 domain (MyTH4), Band 4.1-ezrin-radixin-moesin domain (FERM), and Src homology 3 domain (SH3), followed by another repeat of MyTH4 and FERM domains (https://www.uniprot.org/uniprot/Q13402). Functions of the MYO7A domain are briefly described in the Table 1. Sensory defects and USH1B are often caused by mutations in human MYO7A protein, which is essential for normal vision and hearing.^7^ MYO7A protein forms a dimer for movement on single actin filaments.^8^ In the N-terminal region of MYO7A protein, a conserved motor domain is present. MYO7A acts as an actin-activated, plus-end-directed myosin protein.^8^ Motor function of MYO7A protein is affected by mutations in the head domain.^9^ In the presence of actin, MYO7A shows ATPase activity; i.e., the residues 158-165 of the motor domain binds to actin and can convert ATP chemical energy into mechanical energy.^7^ The tail domain interacts with MYO7A and Rab Interacting Protein (MYRIP), a vesicle-associated protein suggesting the involvement in cargo transport.^9^ C-terminal proline-rich regions interact with the SH3 domain.^10^ FERM domains are known to interact with various membrane integral proteins and in connecting the cytoskeleton to the plasma membrane of the cell.^11^ Deformation of stereocilia and impairment of hearing can occur when the interaction is weakened or in the presence of mutations of MYO7A.^12^

In inherited diseases found in humans, genetic alterations affect the structure, function, and stability of protein globular domains in multiple ways.^13^ These alterations can disrupt the protein energy landscape and result in misfolding of protein molecules.^14^ A precise link between genetic mutations and disease phenotype is difficult to establish. It has also been previously demonstrated that the perturbations caused by genetic mutations modeled at the atomic level of protein structure were associated with patients’ b-wave electroretinogram (ERG) amplitudes, as demonstrated for X-linked retinoschisis.^15,16^ Recently, we developed an unfolding mutation screen (UMS) to evaluate the effect of missense mutations on protein stability.^17–21^ In the UMS method, the effect of the mutation on protein stability is obtained from an unfolding propensity for each possible missense mutation. The propensity is derived from the changes in Gibbs free energy between the wild-type and mutant protein structures. The UMS serves as a tool to analyze the complex relationship among missense mutations, protein folding, and disease.^21^ The global computational mutagenesis or which we use in our work, was verified using experimental data.^17,19,20^

Our unfolding calculations were verified by experiments on 1,391 mutant variants from 16 protein crystal structures. The analysis showed that the proteins from the validation dataset had an average of 77.9±9.1% correct matches between the experimental and computed unfolding. The method was then applied to analyze the mutations and critical residues in genetic eye disorders, such as age-related macular degeneration, autosomal dominant Retinitis Pigmentosa, and Leber’s congenital amaurosis, and others. Here, we created a homology model of MYO7A dimer, refined the structure using molecular dynamics simulations, and applied the global computational mutagenesis to evaluate the severity of genetic disease-related changes at the protein level. To further understand how a defective protein can affect USH1B, we evaluated the effect of each missense mutation on the unfolding properties of the MYO7A protein. Our analysis suggests that the disease-causing effect of pathogenic mutations in inherited diseases such as USH1B could associate with MYO7A protein misfolding. Our *in-silico* study contributes towards the understanding of complex relationships among disease phenotypes, including USH1B involving genetic alterations of MYO7A protein.

## Materials and methods

### Molecular modeling

The protein sequence for *Homo sapiens* MYO7A Isoform1 (residues 1-2,215) was obtained from the UniProtKB accession number (AC) Q13402 (https://www.uniprot.org/ Q13402). MYO7A protein forms a dimer for the movement on single actin filaments. Thus, we performed homology modeling of the residues of MYO7A using a molecular-graphics, -modeling, and -simulation program Yasara.^22^ Several PSI-BLAST iterations within the Yasara tool were used to detect distant sequence similarities via sequence searches. In the next iteration of PSI-BLAST, multiple alignments of the highest-scoring pairs from the first run were generated and a position-specific scoring matrix (PSSM) was calculated from the multiple alignments. The conservation pattern in the alignment was captured in this PSSM and was stored as a score-matrix for every individual position. High scores are assigned to highly conserved positions whereas low or near zero scores are assigned to weakly conserved positions. This profile was used to further perform a database search and identify sequences that complement the PSSM conservation pattern. From the second round of PSI-BLAST searches, the newly identified sequences that are above the e-value cutoff were added to the alignment. Another search was performed, and the process was run until no new sequences were identified that crossed the threshold cutoff. PSI-BLAST iterations inherent to the PSI-BLAST algorithm with a 0.5 maximum PSI-BLAST E-value allowed for templates were employed against the Uniprot database to construct a PSSM from related sequences. Finally, Protein Data Bank (PDB, https://www.rcsb.org/) was searched to identify possible modeling templates by identifying known structures with a similar sequence as Myo7A. Then, models were built for each matched template. To identify a region as reliably aligned, there should be about 65% consensus between different structure alignment methods.^23^ After the first round of structure building was performed, we evaluated the dimeric templates for quality based on Z scores. Quality Z scores are calculated as the weighted average of the individual Z scores using the following formula:

Overall=0.145∗Dihedrals+0.39∗packing1D+0.465∗Packing3D.

A Z score reflects the number of standard deviations away from the average of the given value. A negative Z score is considered poor as it is given to templates that are considered unacceptable when compared to the average high-resolution X-ray structure and when a structure has more outliers in the Ramachandran plot. Next, another round of modeling was performed by providing the top three templates to improve the structure quality. The quality Z scores were again calculated for the resultant dimer. To assess the quality of the model, we ran PROCHECK^24^ and found that 87.7% of the residues are in the most favored regions in the Ramachandran plot.

MYO7A structure was optimized in water using 5 ns molecular dynamics simulation with AMBER14 forcefield with a periodic cell boundary condition. For this purpose, we used “md_runfast” Yasara macro, to run a simulation swiftly at 2*2.5fs time step using the standard macro “md_run”. A cubic shape was selected for the simulation cell extending 2 Å around all the atoms of the structure. The simulation cell with a periodic cell boundary was filled with water at 0.997 g/ml density and 1.4 Å water probe radius. Amber 14 forcefield was used and particle mesh Ewald algorithm with an 8.0 Å cutoff was employed for long-range electrostatics. Simulation was performed at 298K and the macro is set to achieve a pH of 7.4 and 0.9% NaCl concentration. We did not employ pressure control but rather allowed the pressure to adjust according to the above stated parameters. The optimized MYO7A model was compared to a known structure of the human Myosin 7A C-terminal MyTH4-FERM domain (PDB ID: 5mv9) recently determined using protein crystallography. The atomic models were structurally superimposed using the Match Maker module from UCSF Chimera and the root-mean-square deviations of atomic positions (RMSD) were evaluated between homology model for MYO7A and 5mv9 to see differences between the models.

### Global computational mutagenesis

To analyze and identify the relationships between protein folding, genetic alterations, and disease phenotype, we used unfolding mutation screen (UMS).^17,21^ We subjected the domains of MYO7A to UMS and generated unfolding propensities and free energy changes (ΔΔG) for any possible missense mutation. The quality of the protein structures built using homology modeling and refined using molecular dynamics simulations was evaluated using a procedure called an “internal control.” This procedure was suggested in our previous work and verified for different proteins.^17,20^ Briefly, this procedure analyzes the overall quality of the side chain rotamers in the homology model by mutating each residue from the protein sequence to the identical residue during a deep mutation scan. In the procedure, the free energy changes are calculated and converted to unfolding propensities for each identity mutation. The quality of the homology model was then determined by calculating the mean, standard deviation, p-value, and 95% confidence interval for the unfolding propensity values calculated over the list of identity mutations.^17^ In this analysis, we searched for small confidence intervals centered on 0.5 with small p-values (~10–16). This procedure was implemented for different frames of molecular dynamics equilibrations to select the best homology model with the lowest overall variation in unfolding propensities.^21^ Homology model and global mutagenesis results for MYO7A are available through the ocular proteome website (https://neicommons.nei.nih.gov/#/proteome). The website contains the following downloadable data: the human MYO7A homology model, heat maps, unfolding propensities, and foldability parameters for any amino acid residue from the protein sequence.

### Disease-related mutations

The critical residues found in global mutagenesis would be most likely the ones that are also associated with the disease. To verify this assumption, we searched the missense mutations that occur in MYO7A and are related to USH1B, sensorineural hearing loss, and deafness found in the Human Gene Mutation Database (HGMD), Professional 2019.4, (http://www.hgmd.cf.ac.uk/ac/gene.php?gene=MYO7A) and ClinVar (https://www.ncbi.nlm.nih.gov/clinvar/). We screened HGMD for all missense mutations that occur in MYO7A and found 213 single-nucleotide mutations associated with genetic disease. Thirty-five mutations that resulted in protein termination were excluded as a part of this study, because we were interested in evaluating the effects of mutations in protein unfolding and not premature protein termination. In ClinVar database, we found a total of 530 missense mutations, out of which 2 were excluded as they were indels. Both the average unfolding free energy change and the average unfolding parameter over the MYO7A domains were calculated.

## Results

### Molecular modeling

To understand the relationship between inherited disease and perturbations at the atomic level of protein structure, we modeled the atomic structure of MYO7A, currently not available from crystallographic studies. Using homology modeling, all structural domains were built and composed in a structure of MYO7A and the dimeric structure of MYO7A protein was generated (Figure 1B). The modeling was done in two steps: first, we ran the software to generate *de-novo* templates for building a quality structure. The dimeric templates were evaluated for their overall Z scores and the structure quality, and then we proceeded with the second round of structure quality based on Z scores. After this, the three best dimeric templates were chosen from the first run to improve the quality of the dimeric structure. There was an improvement seen in quality of the MYO7A dimer produced in the second round (with a Z score of −0.388) as compared to the original three templates provided in the first round (Z scores of −0.838, −0.802, and −0.813). After performing molecular dynamics simulations in AMBER14 forcefield, we performed homology modeling using two chains of MYO7A to mimic the cargo-carrying behavior. The dimeric homology model, consisting of two identical chains comprising 2,215 residues each of MYO7A, is shown in Figure 1B. We compared the MyTH4-FERM domain model to a published X-ray diffraction structure (PDB ID: 5mv9). We ran iterative pruning in UCSF Chimera to remove residue pairs (which are far apart from each other) using a default cut-off value of 2 Å. After removing 109 pruned atom pairs, we found that the RMSD value between the superimposed structures is 1.2 Å. These results indicate that 109 of the residues are in a different conformation compared to the X-ray diffraction structure of 5mv9. Thus, our comparison confirmed the reliability of the C-terminal domains in our MYO7A model. Therefore, the model was further used to understand the effect of genetic mutations of MYO7A and its role in USH1B.

### Global computational mutagenesis and disease-related variants

**Figure 2:**
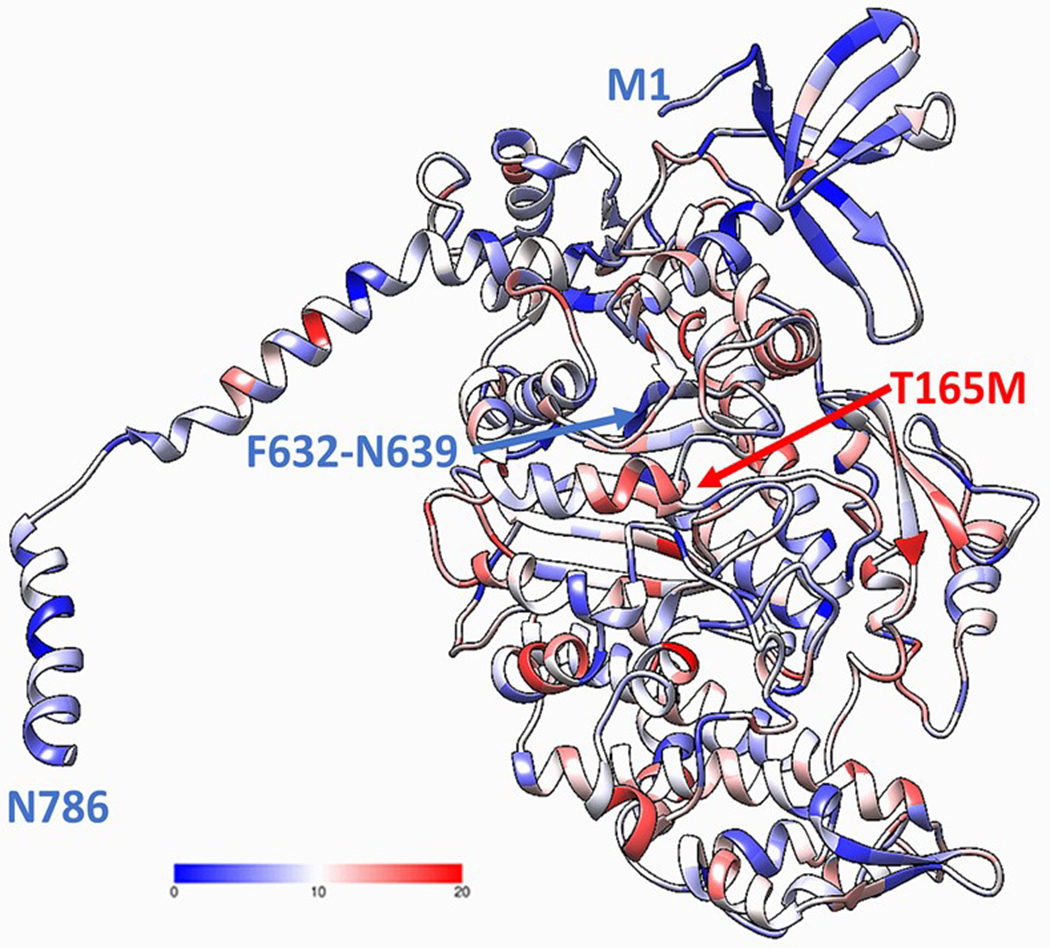
The fragment of MYO7A homology model showing the motor domain (residues 1-786) obtained using homology modeling and global mutagenesis. Protein domains are colored using foldability parameter, protein unfolding was evaluated from global computational mutagenesis (17). The foldability parameter changes from 0-20 and is shown by the colored bar. The maximum value of the foldability parameter (red) corresponds to the residues critical for protein stability when mutated to any amino acid (18, 19). Low foldability (dark blue) corresponds to residues, which are not critical for protein stability.

**Figure 3:**
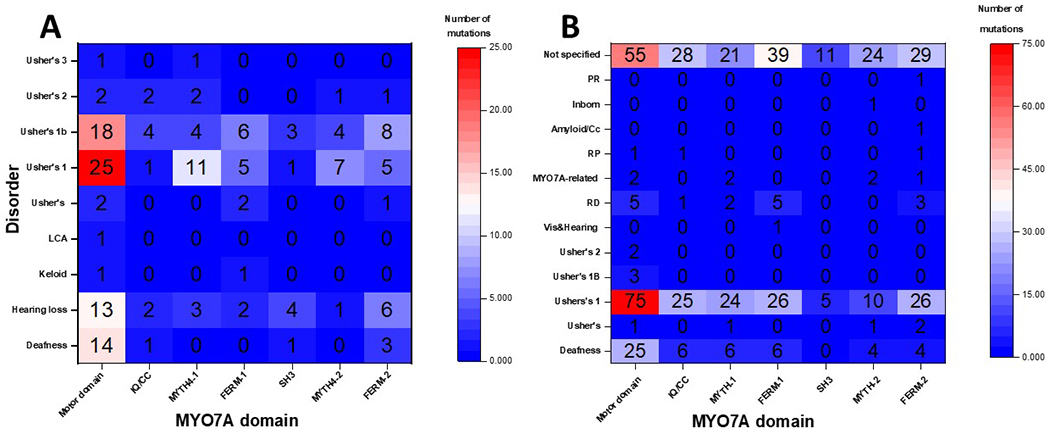
Heatmaps presents the relationships between the location of missense changes in MYO7A domains and disease phenotypes. (A) A heatmap shows the statistical distribution of HGMD missense alterations over MYO7A structural domains. (B) A heatmap shows the statistical distribution of ClinVar missense alterations over MYO7A structural domains.The numbers of the heatmap correspond to the number of HGMD or ClinVar missense mutations for given structural domain and disease phenotype.

We used global computational mutagenesis to determine parts of MYO7A susceptible to the effect of missense changes. The MYO7A complete foldability pattern is available at the ocular proteome website. Residue foldabilities for any possible single alterations for the motor domain of the MYO7A are shown in Figure 2. This figure shows a ‘potential’ risk due to protein instability for each amino acid (16). Residues 632-639 are involved in the actin binding. Mutation T165M is a pathogenic mutation in ClinVar that is predicted as a severe mutation by global mutagenesis. The severity of other mutations in this site are not defined as pathogenic in ClinVar and are predicted as moderate by the global mutagenesis. Thus, using the foldability patterns and unfolding propensities for the human MYO7A model, HGMD and ClinVar mutations were quantified accordingly. HGMD and ClinVar disease-related missense mutations, which were found in MYO7A, were clustered by the related disease phenotypes and were plotted in a statistical distribution of the number of missense mutations over the domains found in the MYO7A homology model Figure 3(A,B). Of all the identified HGMD mutations, 79 were found in the motor domain, 6 in the IQ repeats, 21 in the MyTH4-1 and 13 in MyTH4-2 domains, 17 in the FERM1 and 24 in the FERM2 domains, and 4 in the SH3 domain. From the various categories of the related disease associated with each of the missense mutations, 19 mutations result in deafness, 29 in hearing loss, 2 in keloid formation, 1 in Leber congenital amaurosis (LCA), 5 in Usher syndrome, 57 in Usher syndrome 1 (US1), 46 in Usher syndrome 1b (USH1B), 8 in Usher syndrome 2 (US2), 1 in malignant melanoma, and 1 in Usher syndrome 3 (US3).

In the Myosin motor domain, the highest number of missense mutations (28 out of 77) result in US1, followed by 19 in USH1B, 14 in deafness, 13 in hearing loss, 2 in the US, 1 each in keloid formation, LCA, and the US2 Just like in the Myosin motor domain, the highest number of missense mutations result in US1 in the MyTH1 and MyTH2 domains, while in the IQ repeats/coiled-coil region, FERM1, and FERM2 domains, the highest number of missense mutations associate with USH1B disease phenotype. The four mutations in the SH3 domain result in four different disease phenotypes. Using ClinVar database we identified 528 missense mutations. 169 mutations were found in motor domains, 61 in the IQ/coiled coil region, 56 in MyTH4-1 and 42 in MyTH4-2 domains, 77 in FERM-1 and 68 in FERM-2, and 16 in SH3 domains. 78 mutations in the motor domain, 25 in IQ/coiled-coil, 24 and 10 in MyTH4-1 and 2, 26 in both FERM-1 and 2, and 5 in SH3 domains corresponds to US1/USH1B. Overall, 51 mutations correspond to rare genetic deafness/deafness, 5 for US, 2 for US2, 1 for visual and hearing impairment, 16 as retinal dystrophy, 7 for MYO7A-related disorders, 3 for retinitis pigmentosa, 1 each for Amyloidosis, Inborn genetic diseases, and Pigmentary retinopathy. However, the conditions for 207 out of the 528 mutations are reported as not provided/not specified.

**Figure 4:**
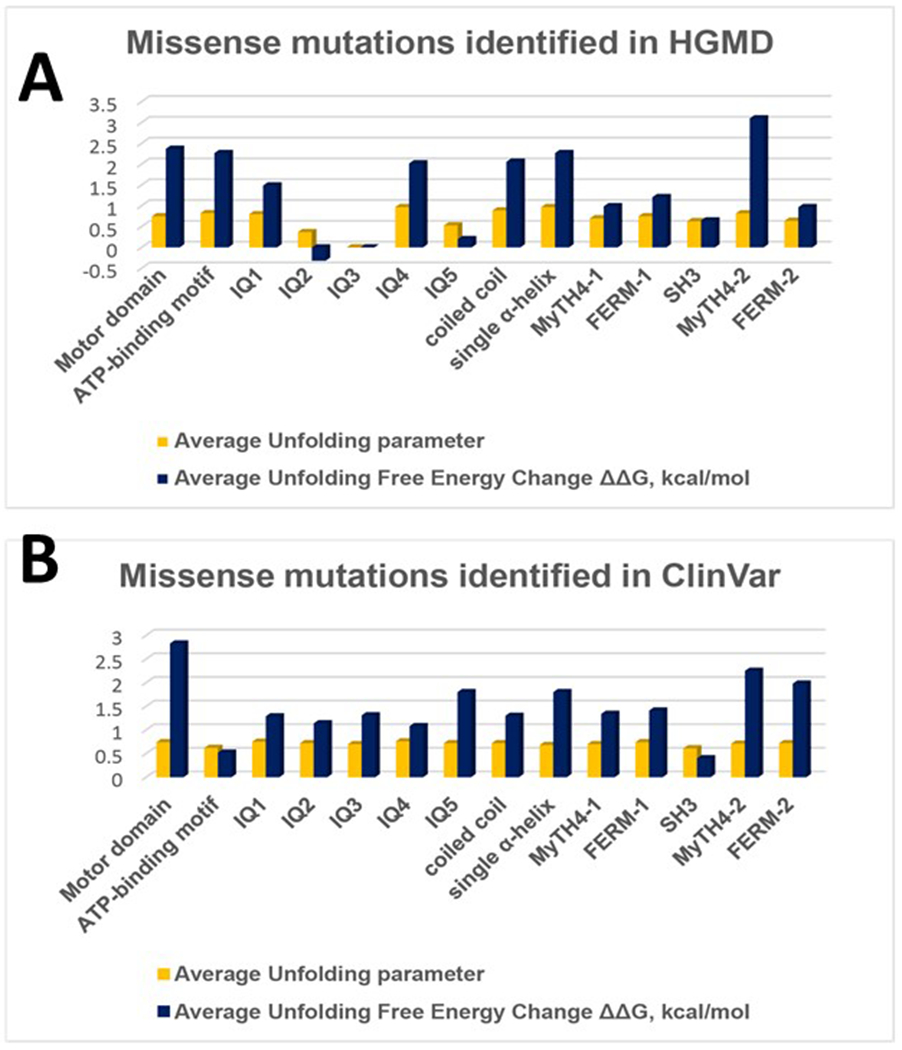
The statistical distribution of averaged unfolding propensities and averaged free energy changes of missense mutations per each MYO7A domain. (A) Missense mutations identified in HGMD. (B) Missense mutations identified in ClinVar.

Per each mutation, the relationship between the mutation effect on unfolding propensity and free energy change is nonlinear (16). Therefore, we used both parameters for the evaluation of the potential mutation effect for each protein domain. Results on the average effect of HGMD and ClinVar mutations on structural domains of the human MYO7A homology model shown in Table 2. Surprisingly, the averaged unfolding propensities and averaged free energy changes correlated for each domain with Adj. R^2^=0.69 for the mutations for HGMD mutations. However, ClinVar showed no correlation between propensities and free energy changes (Adj. R^2^=0.35). We further plotted the average unfolding free energy change and the average unfolding parameter over the MYO7A domains Figure 4(A,B). Both the motor domain and the ATP-binding region in the motor domains showed an average unfolding parameter (0.75, 0.72) with the corresponding average unfolding free energy changes (2.6, 1.4 kcal/mol). These parameters are associated with the highest number of missense mutations from US1 and USH1B disorders. Mutations in the ATP-binding region of the motor domain cause severe effects in the stability of the protein and lead to deafness, US1, and USH1B.

In the neck region of MYO7A, IQ repeats and the coiled-coil region (0.7, 0.82) showed an average unfolding parameter with the average free energy change of 1.08, 1.86 kcal/mol, respectively. We found 8 ClinVar missense mutations in IQ3 but none in the HGMD database. However, IQ2 and IQ5 had a lower average unfolding parameter (0.37, 0.53) and subsequently lower average free energy changes (−0.32, 0.2 kcal/mol) but the corresponding was not true in ClinVar. These differences in the neck region may potentially arise from the fact that a residue unfolding change could either lead to destabilization of the entire protein as the two chains of the protein are bound at the neck region or allow for some flexibility to enable cargo transport without uncoiling. Most of the mutations in this region are associated with US1/USH1B.

In the MyTH4 domains, 1 and 2, the average unfolding parameter was 0.7 and 0.77 with average energy changes 1.17 and 2.68 kcal/mol, respectively, with most mutations also leading to US1/USH1B. On the other hand, in the tail region, the FERM domains (1 and 2) also associate with US1/USH1B and the mutations in this region result in 0.75 and 0.68 average unfolding parameters and 1.31 and 1.48 kcal/mol average free energy changes, respectively. SH3 domain shows average statistics, 0.62 unfolding parameter, and 0.525 kcal/mol average free changes, in most mutations that lead to hearing loss and USH1B. Overall, most mutations in the 3D structure of the MYO7A protein are known to associate with USH1B with an entire protein average unfolding parameter of 0.7 and an average unfolding free energy change of 1.42 kcal/mol (Table 2).

## Discussion

Usher syndrome type 1B (USH1B) is a genetic disorder caused by mutations in the unconventional Myosin VIIa (MYO7A) protein. Homology modeling of MYO7A helps to understand the complex relationships between disease phenotype and protein structure in inherited eye diseases like USH1B. Because of the shortage of computational and experimental methods, protein structure predictions for large proteins has been difficult. USH1B is a disorder associated with a hearing loss due to abnormalities in the inner ear and vision loss due to retinitis pigmentosa. Here, we built and refined the homology model of MYO7A dimer (Figure 1B). Global computational mutagenesis was applied to evaluate the effect of missense mutations crucial for protein structure and stability. The MYO7A structure was applied for the evaluation of the severity of 706 (178 HGMD+528 ClinVar) missense mutations available for the disease and correlated these changes with the disease phenotype. The model can be used to study the interaction with other small molecules, drugs, and ligands to help in a study of the complex relationships among disease phenotypes in diseases that affect the eye.

The shortage of experimental data and bioinformatics methods has hampered the structure prediction of large multidomain proteins. Computational modeling of multidomain structures is complicated, and very few methods are available for this approach.^25^ Potentially, homology modeling of proteins can produce a high-resolution protein atomic structure.^26^ We used the Yasara *de-novo* building feature (Build “residue in a protein”) from the FASTA file to build and assemble the protein structure. Residues were added to the C-terminal end of the MYO7A protein and joined to the adjacent residue via a peptide bond. The structural templates used to produce the final model of MYO7A covered the myosin head and the 4 IQ repeats. The other domains were built using the Yasara *de-novo* building feature due to a lack of a suitable template. In our paper, global computational mutagenesis (16, 19, 20) evaluates the effect of genetic missense mutations on protein structure and stability. Unfortunately, computational forecasting of changes in protein stability and unfolding propensities depends on a significant degree from inaccuracies of molecular modeling for monomeric and oligomeric structures, resolution limits for structural templates, non-stability related changes, like in functional sites, interfaces, post-translation modifications sites, and other parameters. On average, we could better predict severe perturbations (16). As mentioned earlier, an average of 77.9±9.1% correct matches between the experimental and computed unfolding is expected to be evaluated correctly using global mutagenesis. Mutations in MYO7A protein could lead to different disease phenotypes Figure 3 (A,B). The availability of the MYO7A homology model helps in understanding the effects of USH1B disease-related mutations. In the future, it will be interesting to verify our predictions by comparing predictions of deleterious versus tolerated variants based on phenotypic data.

Disease-related mutations can affect the stability of the protein by causing unfolding or protein premature termination. The homology model for MYO7A, colored using unfolding propensities to depict the risk of protein unfolding was evaluated from global computational mutagenesis and is available at the ocular proteome website. We analyzed 706 missense mutations from the HGMD and ClinVar databases in MYO7A. These mutations are related to USH1B, hearing loss, and deafness. Unfolding values associated with these disease-related mutations could predict the stability of domains and, thereby, the protein. About 43.26% (77 out of 178 in HGMD) and 41.9% (221 out of 528 in ClinVar) of the disease-related missense mutations have high destabilizing effects (i.e., unfolding >0.9). Besides, around 52.25% (93 out of 178) and 50.38% (266 out of 528) of the mutations cause a severe effect. The mutations are critical in maintaining the MYO7A protein structure and stability. Other mutations, 252 (51 HGMD+201 ClinVar) disease-related mutations fell under moderate, 46 (30 HGMD +16 ClinVar) as stabilizing, and 53 (4 HGMD+49 ClinVar) under the weak category. Most of these mutations result in Usher syndrome 1 and Usher syndrome 1b (Figure 3).

Human MYO7A is a multi-domain protein carrying numerous functions. Several eye and hearing disease phenotypes are associated with genetic alterations in this protein. A cure for these diseases is related to the further understanding of the link between protein function, genetic alteration, and disease phenotype. A joint effort in protein biochemistry, structural biology, computational mutagenesis, and clinical studies could address such a challenge. To verify the effect of mutations on protein stability, MYO7A must be expressed and purified *in vitro* using recombinant protein technology. Protein expression and purification is a difficult task for large proteins with >2000 residues. Current methods work better for smaller proteins of 50-600 residues, that can be expressed in *E. Coli* (>80% of RCSB). Also, the post-translational modifications for human proteins are not available through *E. Coli* expression systems. Alternatively, the proteins could be modified post-translationally in a larval system.^27–29^ One example is tyrosinase with pathogenic mutations causing oculocutaneous albinism type 1A.^30,31^ Therefore, we hope that biochemical, computational, and clinical validation for a large multi-domain protein like an MYO7A will be possible in the future.

## Conclusion

USH1B is a genetic disorder caused by mutations in the unconventional Myosin VIIa protein (1–2,215 residues). We built the homology model of MYO7A homodimer and refined the structure using 5 ns molecular dynamics in water. Global computational mutagenesis was applied to evaluate the effect of missense mutations that are critical for maintaining protein structure and stability. Motor and MyTH4-2 domains of MYO7A are the most susceptible to pathogenic mutations. Most mutations predicted as causing MYO7A protein misfolding found to associate with USH1 and USH1B. Our analysis suggests that the disease-causing effect of pathogenic mutations in inherited diseases such as USH1B could associate with protein misfolding. Our work contributes to the understanding of inherited disease from the atomic level of protein structure and analysis of the impact of genetic mutations on protein stability and genotype-to-phenotype relationships in human disease.

## Figures and Tables

**Table 1:** Myo7A domains and domain functions

Domain name	Domain function
Motor domain	Movement of MYO7A protein on actin filaments is enabled by the motor domain. The motor domain interacts with ATP and that translates into production of mechanical energy.
IQ1-IQ5 motifs	The CamIQ region regulate the function of motors in a Calcium (Ca2+)-regulated manner and acts as the lever of myosin arms.
Coiled coil	The coiled-coil region assists in the MYO7A dimer formation.
MyTH4 and FERM domains	An N-terminal proline-rich region precedes both MyTH4 and the FERM domains. F1, F2, and F3 lobes are present in each FERM domain. These lobes are known to interact with various membrane integral proteins and in connecting the cytoskeleton to the plasma membrane of the cell.
SH3	SH3 domain helps in recruiting Arp2/3 complexes, which regulates the actin cytoskeleton. C-terminal proline-rich regions of Myo7A can interact with the SH3 domain.

**Table 2:** Summarized effect of missense changes in MYO7A domains

		HGMD			ClinVar		
Domain	Location in domain	Number of missense mutations	Average unfolding propensity	Average free energy change ΔΔG, kcal/mol	Number of missense mutations	Average unfolding propensity	Average free energy change ΔΔG, kcal/mol
Motor domain (excluding ATP-binding region)	65-741	75	0.75	2.37	166	0.74	2.83
ATP-binding motif	158-165	4	0.825	2.27	3	0.62	0.52
Actin-binding	632-639	none	-	-	4	0.62	0.39
IQ1	745-765	2	0.8	1.49	6	0.75	1.29
IQ2	768-788	1	0.37	−0.32	4	0.72	1.14
IQ3	791-811	none	-	-	8	0.7	1.31
IQ4	814-834	1	0.97	2.02	10	0.76	1.08
IQ5	837-857	2	0.53	0.2	4	0.72	1.8
coiled coil (excluding single α-helix)	858-1016	3	0.89	2.06	8	0.72	1.3
single α-helix	858-935	2	0.97	2.27	21	0.68	1.8
MyTH-1	1017-1253	21	0.7	0.99	56	0.7	1.34
FERM-1	1258-1602	17	0.75	1.21	77	0.74	1.41
SH3	1603-1672	4	0.63	0.65	16	0.61	0.4
MyTH-2	1747-1896	13	0.82	3.1	42	0.71	2.25
FERM-2	1902-2205	24	0.64	0.97	68	0.72	1.98
